# Prognostic Significance of BRAF V600E Mutation and CPSF2 Protein Expression in Papillary Thyroid Cancer

**DOI:** 10.3390/biomedicines11010053

**Published:** 2022-12-26

**Authors:** Irena Ivković, Zgjim Limani, Antonia Jakovčević, Srećko Gajović, Sven Seiwerth, Ana Đanić Hadžibegović, Drago Prgomet

**Affiliations:** 1 Department of Otorhinolaryngology-Head & Neck Surgery, University Hospital Centre Zagreb, 10 000 Zagreb, Croatia; 2 Department of ENT-Head & Neck Surgery, University Clinical Center of Kosovo, 10 000 Prishtina, Kosovo; 3 Faculty of Medicine, University of Prishtina “Hasan Prishtina”, 10 000 Prishtina, Kosovo; 4 Department of Pathology and Cytology, University Hospital Centre Zagreb, 10 000 Zagreb, Croatia; 5 School of Medicine, University of Zagreb, 10 000 Zagreb, Croatia; 6 Croatian Institute for Brain Research, School of Medicine, University of Zagreb, 10 000 Zagreb, Croatia; 7 Institute of Pathology, School of Medicine, University of Zagreb, 10 000 Zagreb, Croatia; 8 Faculty of Dental Medicine and Health, University J.J. Strossmayer, 31 000 Osijek, Croatia

**Keywords:** papillary thyroid cancer, metastases, CPSF2, BRAF V600E mutation, recurrence

## Abstract

The early-stage diagnosis of papillary thyroid cancer (PTC) has significantly increased in incidence worldwide without any beneficial impact on survival. In order to improve the risk assessment in PTC, we have conducted a retrospective study in which we analyzed the BRAF V600E mutation and CPSF2 protein expression as prognostic markers on archival tissue samples of 49 patients without (control group) and 97 patients with (study group) PTC metastases in the cervical lymph nodes at the time of initial diagnosis. Our aim was to correlate the BRAF V600E mutation and the expression of CPSF2 protein with the clinical and pathological features of PTC. The expression of CPSF2 protein was evaluated via immunohistochemistry and graded semi-quantitatively. The presence of the BRAF V600E mutation was determined via real-time polymerase chain reac-tion (PCR). CPSF2 protein < 3+ intensity expression was correlated with more frequent recurrences (Fisher-Freeman-Halton exact test; *p* = 0.010; 95% CI: 1.26–22.03), and patients who presented with the BRAF V600E mutation and CPSF2 protein expression < 3+ intensity had shorter disease-free survival (log-rank test; 105.0 months vs. 146.6 months; *p* < 0.001; HR 8.32, 95% CI: 2.91–23.83), whereas patients with PTC who had CPSF2 expression 3+ had longer disease-free survival in correlation with other lower intensity expressions of CPSF2 protein (log-rank test; 139.7 months vs. 129.6 months; *p* = 0.008). The multivariate analysis showed that younger patients with CPSF2 protein expression <3+ and the BRAF V600E mutation are at an increased risk for recurrence and require more intensive monitoring (Cox proportional hazards regression model; X2 = 17.5, df = 10, *p* = 0.025). Our results correlate the BRAF V600E mutation and CPSF2 protein expression with recurrence and disease-free survival as relevant prognostic factors for PTC.

## 1. Introduction

The thyroid is the most common site of cancer in young women between the ages of 15 and 34, with papillary thyroid cancer (PTC) being the most common histological type. There has been a significant increase in reported incidences worldwide in the last 30 years [1,2,3,4], which has resulted in increased interest in identifying potential new prognostic and therapeutic molecular biomarkers for PTC. A high incidence of PTC is attributed to the increased availability of ultrasound and other imaging studies, which is now offered for the preventive and systematic examination of the healthy and “asymptomatic” population. These “preventive” examinations have led to the fact that the total number of patients diagnosed with PTC is of a greater number of those that are diagnosed in the early stages of the disease [1,2,3,4]. Until recently, total thyroidectomy was considered as being the only modality of surgical treatment, followed by radioactive iodine (RAI) ablation (Iodine-131) and thyroid stimulating hormone (TSH) suppression. This approach creates a financial burden on the healthcare system and potentially impairs the health and quality of life of these patients, without a decrease in mortality or improved survival in a disease with an indolent course. Although, the 2015 American Thyroid Association (ATA) guidelines endorse thyroid lobectomy as being a safe and sufficient procedure in selected patients, with a low to intermediate risk of recurrence [5,6,7]; their recommendations are very general and still allow for a different extent of surgery for the same stage of the disease (lobectomy vs. total thyroidectomy). However, they advise to take into consideration several clinicopathological features, including the personal history of radiation treatment to the head and neck, a familial history of thyroid cancer, tumor size, extrathyroidal extension (ETE), and regional or distant metastases and multifocality, prior to surgery [7]. Moreover, the latest recommendations foresee post-operative RAI treatment according to risk stratification for patients with PTC. There is no question as to whether RAI benefits high-risk patients, whereas controversy remains for intermediate-risk and some low-risk patients as per ATA risk stratification [7,8]. Therefore, precisely identifying a small number of patients with a poor clinical outcome would enable a more aggressive therapeutic approach and more frequent and extensive follow-up in these patients, while simultaneously avoiding excessive diagnostic procedures and treatment complications in the majority of patients with an indolent course of the disease. An ATA risk stratification for thyroid cancer is the most widely used risk stratification system, and includes several postoperative clinicopathologic features [7]. Increased efforts have been invested in finding prognostic biomarkers in fine needle aspiration biopsy (FNAB) material, which, along with known clinical parameters and ultrasound features, could be sufficient for performing a preoperative risk assessment.

The BRAF V600E mutation has such a potential. Mutations of the BRAF proto-oncogene play a significant role in cell and tumor progression, with the BRAF V600E mutation being the most common genetic mutation of PTC [9,10,11,12,13]. Numerous studies have established a correlation between the BRAF V600E mutation and the aggressive behavior of PTC [14,15,16,17,18,19], and the BRAF V600E mutation is considered to contribute to the risk of a poor clinical course in the current ATA risk stratification system [7]. The incidence of the BRAF V600E mutation in PTC is reported to be constant or even increasing over the past decades, but most studies emphasize the great variability in the prevalence of the BRAF V600E mutation in PTC, ranging between 30% and 80%, depending on the geographic location and iodine consumption [9,10,11,12,13]. Preclinical evidence suggests that the BRAF V600E mutation significantly reduces the sodium/iodide symporter (NIS) expression and RAI accumulation [20], and therefore, the presence of this mutation is important, not only as a prognostic marker, but also for the subsequent targeted treatment of such PTCs with BRAF inhibitors. Analyzing the genome and gene expression levels in PTC tissue, Nilubol Naris et al. [21] identified five genes (CPSF2, LARS, AURKC, TRNT1, and BCL11A) that are differentially expressed in patients with PTC that are associated with a higher mortality rate. Of the five genes, cleavage and polyadenylation specificity factor subunit 2 (CPSF2) had the highest predictive value. CPSF2 is a 100 kDa sized subunit of the CPSF protein consisting of four polypeptides (160, 100, 73, and 30 kDa), and is required for the cleavage and poly-adenylation of mRNA. A decreased expression of the CPSF2 gene is reported to correlate with the invasiveness of carcinoma cells, the aggressive variants of PTC, and therefore, it is consequently associated with a poor clinical outcome and prognosis [22,23]. PTCs with a decreased CPSF2 protein expression are reported to correlate with a higher rate of metastases in cervical lymph nodes, distant metastases, and recurrence [22,23]. So far, there have not been enough studies to demonstrate that the decreased expression of CPSF2 protein can be used as a sole and independent predictive marker of a less favorable form of the disease when excluding the presence of the BRAF V600E mutation in PTC. Nilubol et al. [22] showed that CPSF2 protein expression is independent of BRAF inhibition: they found no difference in CPSF2 protein expression between untreated cells (control group) and treated mutated PTC cells (study group) with a BRAF inhibitor (vemurafenib). This conclusion further encouraged us to determine the prognostic significance of the combination of these two biomarkers in PTC. We have conducted a retrospective study in which we analyzed the BRAF V600E mutation and CPSF2 protein expression as prognostic markers on archival tissue samples of 49 patients without (control group) and 97 patients with (study group) PTC metastases in the cervical lymph nodes at the time of initial diagnosis. We correlated a BRAF V600E mutation and the expression of the CPSF2 protein with the clinical and pathological features of PTC with the aim of improving the existing risk stratification systems for PTC.

## 2. Patients and Methods

### 2.1. Participants

Informed consent was obtained, and the Ethics Committee of University Hospital Centre Zagreb (class 8.1-16/1-2, Number 02/21 AG; approval issued on 22 February 2016) approved this study under the Helsinki Declaration guidelines, most recently amended in 2013.

This study was retrospective. Samples of PTC and metastatic PTC were obtained from the archives of the Department of Diagnostic Pathology and Cytology, University Hospital Centre Zagreb (UHC Zagreb), collected from patients that had been surgically treated at the Department of Otorhinolaryngology—Head & Neck Surgery, UHC Zagreb, between 9 January 2000 and 10 January 2014. The estimated sample size for the expected difference in the average survival rates was 20% (log rank test) with α = 0.05, and the sample power of 80% was 134 subjects.

We included 150 patients, 50 without (control group) and 100 with (study group) metastases in cervical lymph nodes at the time of initial diagnosis, treated by a single surgeon. All patients had undergone preoperative ultrasound examination, and FNAB of suspicious thyroid nodules and cervical lymph nodes, and were diagnosed with PTC or metastatic PTC. The diagnosis was postoperatively confirmed through histology.

The exclusion criteria were previous head and neck surgeries or irradiation, and the minimal surgical procedure was total thyroidectomy. All patients in the study group also underwent selective neck dissection (level VI or II–V, and VI). We included only patients for whom detailed pathohistological data were available (TNM classification and staging according to the American Joint Committee on Cancer (AJCC) and the Union for International Cancer Control (UICC) [24], histological variant, presence of lymphovascular invasion, multifocality, presence of extrathyroidal extension or cervical lymph node metastasis). The minimal required follow-up period was 5 years. Recurrence was defined as the first clinical return of PTC, i.e., increased serum thyroglobulin (Tg) and tumor presence confirmed by imaging studies, biopsy, or surgery during the follow-up period. It included local relapses in thyroid bed, lymph node metastases, and distant metastases. We consider all patients with increased Tg values and active focuses of the disease that do not accumulate RAI to be RAI-resistant.

In total, samples of 49 patients without (control group) and 97 patients with (study group) metastases in the cervical lymph nodes at the time of initial diagnosis were analyzed. The study group was further divided into three groups as follows: patients with metastases present only in level VI (central), patients with metastases present in level VI (central) and the levels II–V (lateral), and patients with skip metastases in the levels II–V (lateral), defined as the absence of metastases in level VI (central).

### 2.2. Hematoxylin–Eosin Staining

Tissue samples were fixed for 24 h in 10% buffered formalin immediately after resection, dehydrated in ethanol and embedded in paraffin, cut into 5 µm thin sections, and stained with a standard method (hematoxylin–eosin, H&E) for light microscope analysis. H&E staining of tissue samples was used to assess the morphological structure and to determine the percentage of tumor cells. Representative paraffin blocks were then selected and cut for further immunohistochemical and polymerase chain reaction (PCR) analysis. The histopathology and immunohistology of PTC specimens were reviewed by two experienced pathologists.

### 2.3. Immunohistochemistry

**Figure 1:**
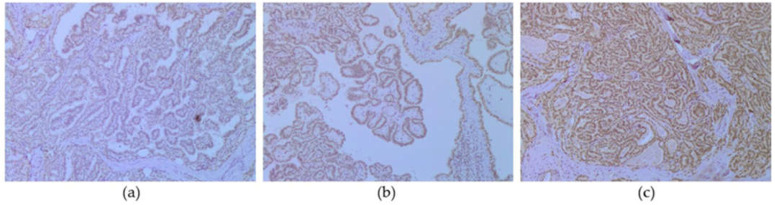
CPSF2 protein expression. (**a**) CPSF2 1+, weak (CPSF2, ABC, ×100), (**b**) CPSF2 2+, moderate (CPSF2, ABC, ×200), (**c**) CPSF2 3+, strong (CPSF2, ABC, ×100).

From the selected paraffin blocks, 4 µm thick sections were obtained for standard immunohistochemical (IHC) staining using the immuno-peroxidase avidin–biotin method with a primary polyclonal anti-CFSP2 antibody (dilution 1:100, Abcam, Cambridge, UK) and automatic stainer (Autostainer, Dako, Glostrup, Denmark). ICH analysis was performed with the EnVision™FLEX (K800 Dako, Glostrup, Denmark) detection system in the above-mentioned tertiary referral center. Nuclear staining intensity was graded semi-quantitatively, using a light microscope, as follows: 0, negative; 1+, weak; 2+, moderate; 3+, strong (Figure 1).

### 2.4. Polymerase Chain Reaction

After determining and marking the tumor and the percentage of tumor cells on the H&E slides, a macrodissection was performed, and 1–5 10 µm sections were obtained (depending on the size of the sample), from which DNA was isolated at the Laboratory for Molecular Pathology University of Zagreb School of Medicine and CHC Zagreb. Isolation was performed using the cobas^®^ DNA Sample Preparation Kit (Roche Holding AG, Basel, Switzerland), and the concentration was measured using a NanoDrop ND-1000 spectrophotometer (NanoDrop Technologies, Wilmington, DE, USA). The presence of the BRAFV600E mutation was detected via real-time PCR using the cobas^®^ 4800 BRAF V600 Mutation Test (Roche Holding AG, Basel, Switzerland) and the cobas z 480 Analyzer (Roche Holding AG, Basel, Switzerland), i.e., the cobas 4800 system, for analysis.

### 2.5. Statistical methods

Data are presented in tables and graphically. In addition to the absolute values and corresponding percentiles, the corresponding 95% confidence intervals were calculated for all categorical variables. The critical z-score value for calculating the 95% confidence interval was 1.96. Differences in categorical variables were analyzed using Fisher’s exact test, or the Fisher-Freeman-Halton test in cases where the tables were larger than 2 × 2. For individual comparisons of tables larger than 2 × 2, the *p* value of significant pairwise comparisons were additionally presented using the Bonferroni correction for differences that were previously statistically significant. Differences in continuous values (age and tumor size) were analyzed using the Mann–Whitney U test with respect to the non-parametric distribution verified using the Kolmogorov–Smirnov test. Kaplan–Meier curves with associated log-rank tests and a hazard ratio (HR) calculation with a 95% confidence interval were used for survival analysis. A multivariate Cox proportional hazards regression model was used to predict a greater risk of relapse. All *p* values of less than 0.05 were considered as being significant. The software supporting MedCalc^®^ Statistical Software version 20.022 (MedCalc Software Ltd., Ostend, Belgium; https://www.medcalc.org, accessed on 10 January 2021) was used in the analysis.

## 3. Results

The descriptive statistics of the clinical and pathological features of PTCs among the included subjects (Total *n* = 146) are shown in Table 1. The total number of patients was 146, of which 115 (78.8%) were women and 31 (21.2%) were men, yielding a 3.7 to 1 ratio. They most often presented with T3 tumor status—70 (47.9%). A total of 73 (50.0%) subjects had an extrathyroidal extension, 90 (61.6%) had multifocal tumors, and 23 (15.8%) had lymphovascular invasion. N 1b nodal status was the most commonly represented, with a prevalence of 41.1%. Occult metastases were present in 13 (8.9%) subjects, and there were 49 (33.6%) patients without cervical metastases. Extracapsular spread in lymph nodes was present in 37 (38.1%) subjects, and distant metastases in 5 (3.4%). In total, 32 (21.9%) subjects had tumor stage 2, while the most common histological type of cancer was the classic variant in 110 (75.3%) subjects. The median age (interquartile range) was 46.0 (32.0–55.0) years, while the median tumor size was 1.3 (1.0–2.2) cm.

Nuclear staining intensity was graded semi-quantitatively using light microscopy, as follows: 0, negative; 1+, weak; 2+, moderate; 3+, strong (Figure 1).

The BRAF V600E mutation was present in 71 (48.6%) subjects, while CPSF2 protein 3+ expression was present in 56 (38.4%) subjects; there were only 4 (2.7%) RAI-resistant subjects, while most subjects—104 (71.2%)—had a moderate risk according to ATA-risk stratification. Recurrence was observed in 19 (13.0%) subjects (Table 2).

**Figure 2:**
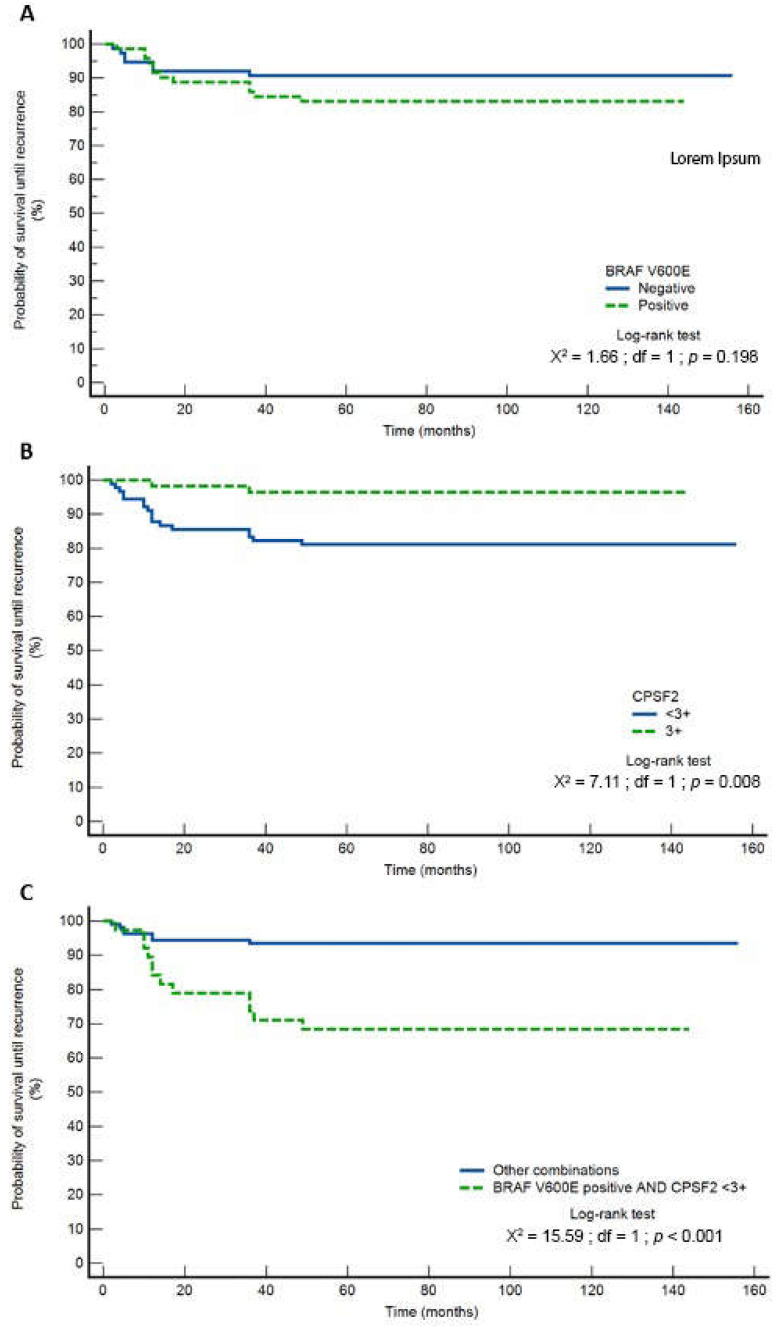
Kaplan–Meier survival curves for disease-free survival in correlation with BRAF (HR of 1.81 (95% CI: 0.73–4.47)) (**A**), CPSF2 groups (CPSF2 < 3+ group—HR of 3.49 (95% CI: 1.39–8.78)) (**B**), and their combination (HR of 8.32 (95% CI: 2.91–23.83)) (**C**).

By comparing the clinical and pathological features of PTC between the subjects with and without metastases, significant differences were found in T status, extrathyroidal extension, multifocality rate, and the occurrence of classic and follicular histological variants. T1a status was significantly more common in subjects without metastases (*p* = 0.021), extrathyroidal extension was significantly more common in subjects with metastases (*p* = 0.009), and multifocal tumors were significantly more common in subjects with metastases (*p* < 0.001), while the classic histological variant was significantly more prevalent in subjects with metastases (*p* = 0.016) and the follicular histological variant in subjects without metastases (*p* = 0.001). There were no significant differences in other observed variables. Subjects with metastases were significantly younger (*p* < 0.001) and had larger tumor sizes (*p* = 0.002). In the group of subjects with metastases, CPSF2 protein 2+ expression was more prevalent (*p* = 0.007), ATA risk assessment was more frequently moderate or high (*p* < 0.001) whereas disease recurrence was more frequent (*p* = 0.004) in comparison to patients without metastases. Subjects with the BRAF V600E mutation had significantly more frequent occult metastases (*p* = 0.008), classic PTC variant (*p* < 0.001), significantly smaller tumor sizes (*p* = 0.021), and a higher rate of CPSF2 3+ expression (*p* = 0.049). In the group with CPSF2 protein expression <3+, lymphovascular invasion was significantly more frequent (*p* = 0.009), as well as recurrence rate (*p* = 0.010). The relative risk (RR) for recurrence in the CPSF2 <3+ group compared with intensity 3+ was 5.29 (95% CI: 1.26–22.03). These data are related only to the number of recurrences without the influence of time, which is later presented in the Kaplan–Meier and Cox regression analysis (Figure 2).

The disease-free interval regarding the presence of the BRAF V600E mutation is presented in Figure 2A. There were no significant differences in survival curves (*p* = 0.198), i.e., the BRAF V600E mutation was not a significant predictor for disease-free interval (log-rank test), and the HR for a group with the BRAF V600E mutation was 1.81 (95% CI: 0.73–4.47). The RR for positive BRAF V600 findings was 1.81 (95% CI: 0.76–4.34; *p* = 0.182), with an actual recurrence rate of 12/71 = 16.9%. It is indicative that after 49 months of follow-up, there were no new changes, i.e., no new recurrences, and the curves were flat. The HR for the CPSF2 < 3+ group was 3.49 (95% CI: 1.39–8.78) (log-rank test—Figure 2B). The RR for CPSF2 <3+ group was 5.26 (95% CI: 1.28–20.0, *p* = 0.022), with an actual recurrence rate of 2/54 = 3.7%.

The group that presented with a combination of BRAF V600E mutation and CPSF2 protein <3+ expression had a HR of 8.32 (95% CI: 2.91–23.83) (log-rank test—Figure 2C). The RR for this combination was 4.87 (95% CI: 2.07–11.46, *p* = 0.0003), with an actual recurrence rate of 12/38 = 31.6%.

Multivariate analysis for the recurrence prediction is shown in Table 3. The Cox proportional hazards regression model was statistically significant (X2 = 17.5, df = 10, *p* = 0.025), and among the predictor variables for the prediction of recurrence, CPSF2 expression <3+ with a HR of 4.97 and 95% CI: 1.08–22.77, *p* = 0.039 stood out as the strongest predictor, while the presence of the BRAF V600E mutation with a HR of 3.82 and 95% CI: 1.04–13.97, *p* = 0.043 was the second strongest predictor. Older age stood out as a protective factor because it reduced the probability of recurrence, with a HR of 0.95 and 95% CI: 0.91–0.99, *p* = 0.020, controlling the influence of all other variables in the model.

## 4. Discussion

The BRAF V600E gene mutation as the most common genetic alteration in PTCs is associated with aggressive behavior and RAI resistance, and hence, a higher rate of recurrence [7,9,10,11,12,13,14,15,16,17,18,19,20,25,26,27,28,29]. So far, there have been no data on the prevalence of the BRAF V600E mutation in patients with PTC in Croatia. The BRAF gene mutation is significant for risk stratification, as well as for potential targeted treatment with BRAF inhibitors in PTC. The BRAF V600E mutation was found in 71 (48.6%) patients in our study. These patients had significantly more often classic PTC histological variants, tumors that were smaller in size, a higher rate of occult metastases, and a higher frequency of CPSF2 3+ expression. In accordance with previous reports, the BRAF V600E mutation status differed significantly within PTC histological variants in our study (*p* < 0.001). We found no BRAF V600E mutation in the solid, oncocytic, clear cell, and diffuse sclerosing variants of PTC in our study. The BRAF V600E mutation correlated with classic, follicular, tall-cell, and Whartin-like PTC histological variants in our study. The results of previous studies on the association between the BRAF V600E mutation and tumor size are contradictory; however, this can also be attributed to different statistical methods and the interpretations of the results. A large meta-analysis by Li C et al. [14] including 32 studies that correlated with the BRAF V600E mutation with clinicopathological features of PTC, as well as a large cohort univariate study from a single center in China [25], have found a significant correlation between the presence of the BRAF V600E mutation and small-sized tumors. In our study, the median size of tumors with the BRAF V600E mutation was 1.1 cm. Metastases to the central compartment (level VI) were statistically more common among tumors with the BRAF V600E mutation than skip metastases or metastases to the lateral neck levels (II–V). We report no correlation between metastases and BRAF V600E status. Joo et al. [25], in their prospective study, also noted that the BRAF V600E mutation was statistically significantly associated with occult metastases in the central compartment (35% versus 15% in the case of the wild type). Given that the presence of the BRAF V600E mutation was determined preoperatively via FNAB, the authors concluded that preoperative analysis of BRAF status via FNAB and tumor size assessment using ultrasound can help to predict occult metastases in the central compartment in patients with PTC with a clinically negative (N0) neck. We also report significantly more frequent occult metastases in patients with PTC harboring the BRAF V600E mutation. This finding can have important implications in tailoring therapy. Based on a preoperative determination of tumor size (T status), multifocality, and the BRAF V600E mutation, elective central neck compartment dissection can be advocated for high-risk patients. Sung et al. [23] reported a statistically significant correlation between the PTC and the BRAF V600E mutation, and CPSF2 protein expression. In a large meta-analysis that included 2167 patients, the BRAF mutation had a sensitivity of 65% in identifying those with recurrence, but it had a positive predictive value of only 25% in predicting the risk of disease recurrence. These findings are in accordance with our results and support our conclusion that the BRAF V600E status alone is not sufficient to contribute significantly to the improvement of risk stratification in most patients with PTC. CPSF2 protein expression in our study was 0 in 1 (0.68%) subject, 1+ in 34 (23.29%), 2+ in 55 (37.67%) subjects, and 3+ in 56 (38.36%) subjects, and these results are comparable with those in the literature. Lower levels of CPSF2 expression in the invasive area of PTC were reported in the literature when correlated with the central part of the tumor; however, this difference was not statistically significant [22]. In support of this finding, our study reports a correlation between group CPSF2 < 3+ expression and lymphovascular invasion. Decreased CPSF2 protein expression (0 and 1+) in PTC has been reported to be associated with a higher rate of metastases in the cervical lymph nodes, and distant metastases and recurrence; thus, with a poor outcome and prognosis [22,23]. Contrary to previous studies, our study group with cervical lymph node metastases showed significantly higher moderate CPSF2 protein expression (2+), while we did not find a correlation between the pattern of cervical metastasis and CPSF2 status. The control group without cervical metastases had a significantly higher prevalence of positive (2+ and 3+) CPSF2 protein expression (71.4%). CPSF2 protein 3+ expression was more prevalent in the group with cervical lymph node metastases (49.0%) compared with the control group without metastases (33.0%). Although the difference was not statistically significant, if we were to increase the number of subjects in the study, the expression of CPSF2 protein 3+ could have a “protective” effect. Sung et al. [23] univariately demonstrated that a tumor size ≥4 cm (2.72(1.07,6.92) *p* = 0.036) and a negative CPSF2 expression (0 and 1+) were significant predictors for regional metastasis, with a tumor size ≥ 4 cm being more significant, whereas in the multivariate analysis, negative CPSF2 expression (0 and 1+) remains the only predictor of regional metastases. We consider that there is a methodological problem in their analysis, since their multivariate analysis included all potential predictors of cervical lymph node metastasis from their univariate analysis, regardless of whether they were significant or not.

Disease recurrence is the most important factor influencing morbidity in PTC, as it results in an impaired quality of life, a risk of repeated surgeries, and exposure to a high cumulative dose of RAI, all of which lead to an increased risk of complications, morbidity, and death [7,26,29]. In our study, the general recurrence rate was 13%, while the study group as opposed to the control group was statistically significantly associated with recurrence. Our research also shows that a CPSF2 expression <3+ has significantly more frequent disease recurrence, which leads us to conclude that these patients require more intensive monitoring. In an in vitro study, Nilubol et al. [22] confirmed that CPSF2 mRNA expression levels in all samples were significantly lower in patients with multiple recurrence episodes, and that CPSF2 mRNA expression levels were associated with a disease-free survival. There were no significant differences in the curves when we analyzed disease-free survival in correlation with the BRAF V600E mutation, i.e., the BRAF mutation was not a significant predictor for disease recurrence. Disease-free survival was significantly longer for the group with CPSF2 expression 3+ (139.7 months versus 129.6 months for the CPSF2 <3+ group; *p* = 0.008). It is indicative that were no new changes after 49 months of follow-up there, i.e., no new recurrence episodes, and the curves were flat. This finding can be considered as a recommendation for a new follow-up protocol based on CPSF2 protein expression. Several studies have reported that the BRAF V600E mutation in PTC correlates with a higher rate of cervical lymph node metastases, multifocality, extrathyroidal invasion, younger age, and a higher rate of recurrence [11,12,14,15,16,17,18,19,20,27,28,29]. The results of our multivariate analysis support additional certainty in predicting recurrence, i.e., younger patients with CPSF2 expression <3+ and the BRAF V600E mutation are at an increased risk of recurrence and require more frequent follow-up. However, we cannot confirm Sung et al.’s [23] finding that the CPSF2-negative group (expression 0 and 1+) had a statistically significant shorter rate of disease-free survival compared with the CPSF2-positive group (2+ and 3+), nor can we concur that CPSF2 protein expression 0 and 1+ is independently associated with a poor clinical outcome in patients with PTC. Although our obtained results do not quite align with their findings, we did find that the group with the BRAF V600E mutation and CPSF2 protein expression <3+ had significantly shorter disease-free survival (105.0 months versus 146.6 months). This is considering that ATA risk stratification already encompasses the BRAF mutation, and we believe that including CPSF2 expression in the risk assessment can improve the current guidelines and stratification system.

We emphasize a relatively short mean follow-up time to detect distant metastasis and PTC recurrence as the main shortcoming of our study. The BRAF V600E mutation and CPSF2 protein expression correlation with the clinically most significant entity—RAI-resistant PTC—is also limited by our sample size due to the very rare occurrence of this entity. We find the same limitations when observing other seldom features such as some tumor variants, distant metastases, or skip metastases. The prevalence of the BRAF V600E mutation in PTC depends on the geographic location and on iodine consumption. Croatia is a small country of great diversity in geography, climate, dietary habits, and iodine consumption, and in this study, we only observe patients that underwent total thyroidectomy in our tertiary referral center, but we are projecting conclusions on the entire population. A retrospective study design and single center experience can also be considered as being limiting factors.

## 5. Conclusions

The BRAF V600E mutation and CPSF2 protein expression are relevant prognostic markers for PTC. Our study results confirm a previous finding of correlation between the BRAF V600E mutation and occult metastases. Not only can the preoperative determination of tumor size in conjunction with BRAF V600E mutation analyses via FNAB assist in predicting occult central lymph node metastasis in patients with clinically node-negative (N0) neck, but we can consider the elective dissection of the central neck compartment in high-risk patients with multifocal PTC. Younger patients with the BRAF V600E mutation and CPSF2 expression <3+ are at increased risk for recurrence, the same as patients with only the finding of CPSF2 expression <3+. Nevertheless, we conclude that a decreased expression of the CPSF2 protein cannot be used as a sole and independent predictive marker of a less favorable form of disease. CPSF2 protein expression can be considered as being a prognostic marker in risk assessment, and can substantially improve guidelines for follow-up protocol and current stratification systems.

## Figures and Tables

**Table 1:** Descriptive statistics of clinical and pathological features of PTC among included subjects at the time of diagnosis (Total *n* = 146).

		*n*	%	95% CI	
Gender	Male	31	21.2%	15.2%	28.4%
	Female	115	78.8%	71.6%	84.8%
Age groups	<40 years	57	39.0%	31.4%	47.1%
	41–60 years	68	46.6%	38.6%	54.7%
	>60 years	21	14.4%	9.4%	20.8%
T status	1a	29	19.9%	14.0%	26.9%
	1b	26	17.8%	12.3%	24.6%
	2	19	13.0%	8.3%	19.2%
	3	70	47.9%	39.9%	56.0%
	4a	2	1.4%	0.3%	4.3%
Extrathyroidal extension	Non-invasive tumors	73	50.0%	42.0%	58.0%
	Invasive tumors	73	50.0%	42.0%	58.0%
Multifocal tumors	No	56	38.4%	30.8%	46.4%
	Yes	90	61.6%	53.6%	69.2%
Lympho-vascular involvement	No	123	84.2%	77.7%	89.5%
	Yes	23	15.8%	10.5%	22.3%
*n* status	0	49	33.6%	26.3%	41.5%
	1a	37	25.3%	18.8%	32.8%
	1b	60	41.1%	33.4%	49.2%
Occult metastases	No	133	91.1%	85.7%	94.9%
	Yes	13	8.9%	5.1%	14.3%
Metastases—region	No regional metastases	49	33.6%	26.3%	41.5%
	Lateral	45	30.8%	23.8%	38.6%
	Region VI	39	26.7%	20.0%	34.3%
	Skip metastases	13	8.9%	5.1%	14.3%
Extracapsular spread	No	60	61.9%	52.0%	71.1%
	Yes	37	38.1%	28.9%	48.0%
Distant metastases	No	141	96.6%	92.7%	98.7%
	Yes	5	3.4%	1.3%	7.3%
Tumor stage	1	114	78.1%	70.9%	84.2%
	2	32	21.9%	15.8%	29.1%
Histological variants	Classic	110	75.3%	67.9%	81.8%
	Follicular	18	12.3%	7.7%	18.4%
	Tall-cell	3	2.1%	0.6%	5.4%
	Solid	4	2.7%	0.9%	6.4%
	Oncocytic	2	1.4%	0.3%	4.3%
	Warthin-like	2	1.4%	0.3%	4.3%
	Clear cell	1	0.7%	0.1%	3.2%
	Diffuse sclerosing	6	4.1%	1.7%	8.3%

**Table 2:** Descriptive statistics for BRAF V600E mutation, CPSF2 expression, RAI resistance, ATA—risk stratification, and the observed recurrence among included subjects (*n* = 146).

		*n*	%	95% CI	
BRAF V600E	Negative	75	51.4%	43.3%	59.4%
	Positive	71	48.6%	40.6%	56.7%
CPSF2 groups	<3+	90	61.6%	53.6%	69.2%
	3+	56	38.4%	30.8%	46.4%
Combination	Other combination	108	74.0%	66.4%	80.6%
	BRAF V600E positive AND CPSF2 <3+	38	26.0%	19.4%	33.6%
RAI resistant	No	142	97.3%	93.6%	99.1%
	Yes	4	2.7%	0.9%	6.4%
ATA	Low risk	17	11.6%	7.2%	17.6%
	Moderate risk	104	71.2%	63.5%	78.1%
	High risk	25	17.1%	11.7%	23.8%
Recurrence	No	127	87.0%	80.8%	91.7%
	Yes	19	13.0%	8.3%	19.2%

**Table 3:** Multivariate analysis for the recurrence prediction: Cox proportional hazards regression model.

	HR	95% CI		*p*
		Lower	Upper	
BRAF V600E positivity	3.82	1.04	13.97	0.043
CPSF2 groups <3+	4.97	1.08	22.77	0.039
Lymphovascular involvement	1.80	0.59	5.49	0.298
Multifocal tumors	1.13	0.34	3.80	0.840
Extracapsular spread present	1.98	0.60	6.46	0.260
Tumor invasion into locoregional tissues	4.40	0.94	20.48	0.059
Metastases—lateral region (ref. value)				0.434
Metastases—region VI	1.87	0.37	9.59	0.451
Metastases—skip	3.35	0.50	22.22	0.211
Age (years)	0.95	0.91	0.99	0.020
Occult metastases	2.83	0.59	13.56	0.192

## Data Availability

The data that support the findings of this study are not publicly available due to information that could compromise the privacy of research participants. They can be made available upon request from the corresponding author.
